# COVID-19, relationships, and contraception: Qualitative perspectives from emerging adults during the COVID-19 lockdown in Accra, Ghana

**DOI:** 10.1016/j.ssmqr.2022.100216

**Published:** 2023-06

**Authors:** Adriana A.E. Biney, Esinam Kayi, D. Yaw Atiglo, Laud R. Sowah, Delali Badasu, Augustine Ankomah

**Affiliations:** a Regional Institute for Population Studies, University of Ghana, Ghana; b School of Continuing and Distance Education, Department of Distance Education, University of Ghana, Ghana; c Population Council, Ghana

**Keywords:** COVID-19 pandemic, Contraception access, Reproductive health, Sexual activity, Youth, Urban family planning

## Abstract

Globally, family planning services were disrupted during the height of the COVID-19 pandemic. Access to these services was a challenge for sexually active urban youth, and this warrants investigation. Using in-depth interview data, we qualitatively explored the effect of the lockdown on the relationship quality and contraception behaviour of emerging adults (19–24 year olds) who were in relationships during a specified lockdown period. Participants were purposively selected from a densely populated urban area in Accra and two public universities in that vicinity. In-depth interviews were also conducted with two family planning providers. Transcripts generated from the interviews were analysed thematically.

Twelve of the 23 emerging adults were sexually active during the lockdown and varied in their reports on the stability of their relationships. The sexually inactive had disruptions in their relationships, mainly due to partner absence and a lack of sexual activity. Modern contraceptives, especially male condoms, were used but were obtained prior to the lockdown as confirmed by family planning providers. Traditional and folkloric methods were used by four participants. Participants reported no unintended pregnancies but rare cases of sexually transmitted infections.

During the height of the COVID-19 pandemic, sexually active urban youth in Accra navigated the restrictions of lockdown imposition with diverse experiences. Therefore, understanding young adults’ unique contraceptive behaviours and practices is essential to providing relevant sexual and reproductive health services to meet their needs. Discussions on the impacts of COVID-19 should be extended to sexual and reproductive health concerns such as access to contraceptives.

## Abbreviations

COVID-19: Coronavirus Disease 19
ECH: Ethics Committee for the Humanities
ECP: Emergency Contraceptive Pill
FGD: Focus Group Discussion
IDI: In-depth Interview
IUSSP: International Union for the Scientific Study of Population
JHS: Junior High School
LaNMMA: La-Nkwantanang Madina Municipal Assembly
MCA: Medical Counter Assistant
SHS: Senior High School

## 1 Introduction

The COVID-19 pandemic and resultant periods of lockdowns, restraints on mobility, migration, local and international trade as well as constraints on health financing at public and private facilities have been associated with various challenges for both the supply and demand for family planning in recent times (Abor & Abor, 2021; Church et al., 2020; Cousins, 2020; Ferreira-Filho et al., 2020; Kenu et al., 2020a; Kumar et al., 2020; Nanda et al., 2020). Indeed, the public health measures and response strategies adopted by governments had adverse implications for sexual and reproductive health, especially that of adolescents and young adults in sub-Saharan Africa (Addae, 2021). Studies acknowledge the effect of lockdown measures on restricted access to essential services, including family planning services at health facilities during the height of the pandemic (Cousins, 2020; Ferreira-Filho et al., 2020; Kumar et al., 2020). The literature also acknowledges issues of inter-couple conflicts as a result of various financial, emotional and social challenges caused by stressors from the lockdown and the pandemic itself (Cousins, 2020). In addition, solutions for addressing restrictions to modern contraception during the pandemic have been proffered (Cousins, 2020; Nanda et al., 2020). However, these recommendations require additional evidence drawn from the experiences of at-risk youth, in order for the recommendations to be better informed, and solutions to be tailored for specific contexts.

Unmarried emerging adults in sexually intimate relationships are an at-risk group prone to reproductive health challenges (Atiglo & Biney, 2018, 2021), with the COVID-19 pandemic further exacerbating these, and this warrants further investigation. These youth may have different challenges during adolescence since they are considered adults; and yet may not have the needed sexual negotiation skills, critical sexual and reproductive health information, self-efficacy, and support to navigate negative relationship experiences and reproductive health encounters as adults.

In sub-Saharan Africa, modern contraceptive use among married and unmarried adolescent girls and young women (aged 15–24 years) is generally low, with a recent study reporting a contraceptive prevalence of 24.7% (Ahinkorah, 2020). The 2017 Ghana Maternal Health Survey reports modern contraceptive prevalence of 27.2% and 39.1% among unmarried sexually active women aged 15–19 and 20–24 years, respectively. Their methods mainly comprise the male condom, injectables, implants, emergency contraceptive pills (ECP) and the oral pill (Ghana Statistical Service (GSS), Ghana Health Service (GHS), & ICF, 2018). Sources of these methods for youth are typically pharmacies, and public and private health facilities, depending on the method (Radovich et al., 2018). Traditional methods, predominantly calendar/rhythm method and withdrawal, are also adopted by young people as 8.4% and 9.7% of those aged 15–19 and 20–24 years, respectively, reported these as their main methods (GSS et al., 2018). This knowledge of youth contraceptive behaviour in Ghana provides an understanding of the general challenges they may face in a pandemic with restrictions to access to their preferred contraceptive methods.

In this study, we used qualitative research techniques to explore emerging adults’ contraceptive use and access during the height of the restrictive period of the COVID-19 pandemic in Accra, Ghana. We aimed to also understand how their intimate sexual relationships were impacted during this restrictive period. We triangulated this information gathered with interviews with family planning providers on their experiences with the provision of family planning services during that same period.

### 1.1 Study context and setting

Ghana reported its first two cases of COVID-19 on March 12, 2020, and experienced rapid community transmission within the first two weeks (Kenu et al., 2020b). Prior to that, the Government of Ghana had instituted a number of response measures as part of a national response strategy to detect, control and prevent lateral community transmission. These mainly included mandatory quarantining of all travelers, social distancing, personal hygiene, restricted mobility, school closures, and bans on public gatherings including educational activities, religious services, social gatherings, and sporting events. On March 30, 2020, the government announced a partial lockdown of Accra and Kumasi, the two largest cities in Ghana with the highest reported cases, in order to enhance surveillance through active case search, contact tracing and hotspot mapping (Afriyie et al., 2020; Kenu et al., 2020b). Although the lockdown was lifted on April 20, 2020, personal hygiene measures, restrictions on public gatherings and school closures were maintained until June 2020 (Addae, 2021; Kenu et al., 2020b).

The main setting for the study was Madina which is the capital of the La-Nkwantanang Madina Municipal Area (LaNMMA). Formerly a peri-urban area outside the Accra Metropolitan Area, LaNMMA has transformed into its own municipality and administrative area under the Greater Accra Metropolitan Area. The municipality and its capital city are densely populated. Due to this, early in the pandemic all COVID-19 restrictions were enforced and monitored due to its susceptibility to becoming a hotspot for infections (Ghanamma, 2020). Madina was a preferred study setting due to the large number of youth migrating to and residing there (Afeadie, 2022; Langevang & Gough, 2009). It is considered an economic hub as it has one of the largest markets in Accra, along with a large transport hub, and several shops and stalls along the various roads in the city centre. The setting provided the study with ready access to informal workers and apprentices. LaNMMA has about 15 educational and research institutions within close proximity making it a “knowledge district”. The two major public universities that undergraduate students were recruited from are located within a 4-km radius of the city centre. All these benefits made this a prime study location.

### 1.2 Theoretical-conceptual framework

The study is underpinned by a series of conceptions such as the social cognitive theory (SCT), as well as stress, coping and health behaviour models, and the psychological stress theory. These models are broadly employed in the study to explain environmental influence, such as restrictions during a lockdown, on health behaviour, including during stressful situations, such as a pandemic (Glanz et al., 2008). Concepts under the SCT help us understand that policies that incentivize certain actions can impinge negatively, positively or may not affect people's subsequent actions (McAlister et al., 2008). For example, a lockdown seeks to reduce the spread of a virus during a pandemic but can disrupt people's way of life or behaviour. Stressful situations resulting from the lockdown and the ways people adopt coping strategies can also influence health behaviour which could involve more risky behaviour (Dodoo et al., 2007; Glanz & Schwartz, 2008).

The psychological stress theory further explains how stress falls under two main concepts: 1) appraisal – comprising “an individual's assessment of the consequence of what is happening to their welfare”, and 2) coping – “an individual's ability to manage the demands of the stress” (Krohne, 2002; Lazarus, 1991). These can easily be related to the effects of the COVID-19 pandemic after the country recorded its first case in March 2020. The stress associated with this was exacerbated when it led to a lockdown in cities later in the month of March, with its associated restrictions and guidelines. For any young person who is in a sexual relationship and is suddenly affected by a lockdown and a series of restrictions (social distancing, avoiding physical contact, school, office or shop closures, and suspensions on trading and travelling) their means of appraising the situation and coping may differ. Thus, it was critical to investigate how the stresses from a lockdown affected their sexual relationships and contraceptive behaviour.

## 2 Material and methods

### 2.1 Study design, recruitment and data collection

We used a phenomenological approach to gather qualitative data for one of the International Union for the Scientific Study of Population (IUSSP) Urban Family Planning Projects focused on exploring the use, non-use, and discontinuation of modern contraception among urban youth in a suburb of Accra, the capital city of Ghana. The larger project comprises data from 30 in-depth interviews (IDIs) and 10 focus group discussions (FGDs) with sexually active young men and women between ages 18 and 24 years. They were selected from three socio-economic subgroups – tertiary students, informal workers, and apprentices representing those in education, employment, and training. Two interviews with family planning providers were also conducted. Because of funding limitations, the decisions to restrict the entire study's sample to 30 IDIs, 10 focus group discussions and 2 family planning providers were prioritized, and thus the criterion of saturation was not used to determine when interviews were stopped.

The relationship and contraception histories of the youth were obtained from the IDIs while FGD participants reported on societal perspectives relating to relationships, contraception, and induced abortion among youth in their socio-economic subgroups. Family planning providers offered accounts on young peoples’ contraceptive behaviours and access to contraception. The inclusion criteria for study participant selection comprised young men and women between the ages of 18 and 24 years who were sexually active. Sexual activity was defined as the person having ever been in a sexual relationship and engaged in sexual intercourse with at least one partner. Young people were also required to belong to any one of the three socio-economic groupings – tertiary student, informal worker or apprentice – and were willing to be interviewed. We excluded young people below age 18 and above 24 years, those not sexually active, and those who were either not enrolled as an undergraduate student in one of the two public universities in Accra, or not in an occupation deemed to be in the informal sector, or not in an apprenticeship. Young people who fit the criteria but did not work or undergo apprenticeship training in Madina were also not eligible to participate in the study. Family planning providers had to be employees of a pharmacy or private health facility in Madina and had regularly provided family planning services to young people. All others without these characteristics were excluded. For this paper, we solely analysed data from 23 out of the 30 IDIs with emerging adults who were either in casual or regular sexual relationships during the lockdown period of late March to late May^1^ (Kenu, 2020b); alongside two IDIs with family planning/reproductive health service providers. The 23 emerging adults were recruited purposively and comprised young men and women from ages 19–24 years.^2^ They belonged to the three socio-economic groupings indicated above.

The tertiary students were recruited from two public universities in Accra through University listservs, flyers circulated through WhatsApp groups, and in-person. The apprentices and informal sector employees were selected from various locations in Madina. Apprentice and informal worker association leaders provided information on their members, to name a few. The youth could call, text or WhatsApp the research assistant's number and he would screen them based on the study's eligibility criteria of age, sexual activity and socio-economic group status. Those who were approached on face-to-face basis and gave their numbers to the recruitment team were called and screened. All the youth who were eligible and willing to participate in the study were visited by interviewers on the chosen dates and preferred interview location agreed on by the interviewee. The two female family planning providers were purposively recruited from a pharmacy and a private clinic within the same location as the apprentices and informal workers. They were approached and asked about participating in the study. Upon their informed consent, appointment dates for the interviews were given and the interviews were conducted.

The interviews with emerging adults were conducted in English, pidgin English, and a local language (Twi) between December 2020 and January 2021. All the interviewers (three male and three female) were fluent in one of the local languages (Twi) used for the interviews and had prior experience with qualitative interviewing. The interviewers were trained to administer informed consent, protect the privacy of participants and refer participants if any traumatic experiences occurred. Interviewers were only allowed to interview participants of the same sex. All interviewees consented to voluntarily participate in the study and have the conversations audio recorded. The participants were provided with about $4 worth of phone credit as a token of compensation for their time.^3^ The family planning providers were interviewed in February 2021 by one of the female authors trained in qualitative research methods.

The qualitative interview guide for emerging adults was developed by the study team to elicit information on their contraception behaviour within each of their sexual relationships in addition to questions about their relationship conflict, contraceptive use, experiences with negative reproductive health outcomes (unintended pregnancies and sexually transmitted infections) during the specified lockdown period. The interview guide for the family planning providers included questions on the impact of COVID-19 on young people's access to family planning services as well as barriers to their use during the lockdown period.

To ensure credibility of the data collected, the study employed a triangulation of sources and investigators as well as reflexivity during the process (Schwandt et al., 2007). Information from emerging adults and family planning providers were triangulated to enable a comprehensive understanding of family planning access during the period. A research team consisting of four researchers and two scientific advisors developed the interview guides and study methodology. The four researchers trained the interviewers and ensured field work was conducted according to the highest standards. The diverse group of individuals in the research team ensured data collection was devoid of inherent biases regarding the team's approach to the study.

Ethical clearance for the study was obtained from the University of Ghana's Ethics Committee for the Humanities (ECH), protocol number ECH 135/19–20. All the 23 interviews were audio recorded and translated and/or transcribed by the interviewers. They lasted between 20 and 55 ​mins.

### 2.2 Data analysis

One of the authors checked and/or back-translated all transcripts for accuracy. To enhance trustworthiness, three authors read through two of the emerging adults' transcripts, and applied deductive and inductive codes to the text. A coding frame was generated which enabled codes to be applied to all other emerging adults' transcripts while additional new codes were also noted in the coding frame. For this paper, only codes related to their COVID-19 lockdown experiences were analysed and reported. We analysed the transcripts using the principles of codebook thematic analysis (Braun & Clarke, 2022), aided by the qualitative data analysis software, Atlas.ti version 7. Codes were grouped into sub-themes under deductively proffered major themes (relationship quality, sexual activity and contraceptive use and reproductive health outcomes). The two family planning providers' transcripts were read by one of the authors and findings relating to young people's access to contraception, and supply and cost of commodities during the lockdown were elicited to complement the study findings. These are discussed in the ensuing sections.

## 3 Results

The findings presented in the succeeding sections highlight participants' reports on their relationship quality, sexual activity, contraceptive use, and access to reproductive health services as well as negative reproductive health issues faced. The results also report information from family planning providers used to triangulate reports from emerging adults’ experiences regarding access to contraception and family planning services during the lockdown period.

### 3.1 Participants’ characteristics

The twenty-three participants (11 female and 12 male) were in existing sexual relationships but only 12 reported that they were sexually active during the lockdown period. The main contraceptive methods used by these 12 varied within the traditional, folkloric and modern categories. There were seven informal workers, seven apprentices and nine tertiary students. The informally employed young men and women mostly engaged in sales of various items on the streets and in small shops. The female apprentices were seamstresses and hairdressers while the male apprentices were learning mobile phone repairs, shoe making and tailoring. The informal workers and apprentices had varying amounts of formal education ranging from none to senior high education. The majority were in regular sexual relationships with a few in occasional sexual partnerships. Nine participants described their relationship as low quality while 13 reported being in a high quality relationship. Table 1 provides the background characteristics of the study participants.

**Table 1:** Socio-demographic, socio-economic, relationship and contraception information on emerging adults in relationships during the lockdown period.

Participants in Lockdown Relationships	Males	Females	Both	Number
Gender				
Male	100.0	–	52.2	12
Female	–	100.0	47.8	11

Average Age (Age Range)	21.4 (19–24)	22.6 (20–24)	22.0 (19–24)	23

Educational Attainment				
None	8.3	0.0	4.3	1
Primary	0.0	18.2	8.7	2
Junior High School (JHS)	8.3	18.2	13.0	3
Senior High School (SHS)	41.7	27.2	34.8	8
Tertiary	41.7	36.4	39.1	9

Socio-economic Groups				
Informally employed	25.0	36.4	30.4	7
Apprentice	33.3	27.2	30.4	7
Tertiary students	41.7	36.4	39.2	9

Relationship Status				
Regular partnership	91.7	90.9	91.3	21
Occasional partnership	8.3	9.1	8.7	2

Relationship Quality				
Low	58.3	18.2	39.1	9
High	33.3	81.8	56.5	13
Did not say	8.3	0.0	4.4	1

Sexual Activity				
No	41.7	54.5	47.8	11
Yes	58.3	45.5	52.2	12

Contraception Prior to Lockdown^a^				
ECP	100.0	75.0	87.0	20
Condom	63.6	83.3	73.9	17
Withdrawal	81.8	66.7	73.9	17
Calendar/Rhythm	27.3	50.0	34.8	8
Implants	18.2	0.0	8.7	2
Folkloric methods^b^	9.1	0.0	4.3	1
**Total**	**100.0**	**100.0**	**100.0**	**23**

Sexually Active Lockdown Participants	Males	Females	Both	Number
Contraceptive use				
None	14.3	0.0	8.3	1
Withdrawal	0.0	40.0	16.7	2
Calendar/Rhythm	14.3	0.0	8.3	1
Folkloric method (Kyem^c^)	0.0	20.0	8.3	1
Condom	42.9	20.0	33.3	4
ECP	28.6	20.0	25.0	3
**Total**	**100.0**	**100.0**	**100.0**	**12**

a The percentage of respondents using each method is indicated. Multiple methods were mentioned by respondents, hence, the column percentages do not add up to 100%.

b The respondent reported using ‘kyem’, drinking cold water and standing immediately after sex.

c Kyem is a a folkloric method where immediately after sex women squat over a toilet and attempt to squeeze the sperm out of their vaginas.

As shown in Table 1, contraceptive use prior to the lockdown period consisted of multiple methods. The ECP was the most reported contraception, followed by the condom, withdrawal and calendar/rhythm. Other methods were implants and folkloric methods. The twelve participants who were sexually active during the lockdown period used the same methods, with the condom and ECP being most reported.

### 3.2 Impact of lockdown on relationship quality

The study participants reported mixed views on the quality^4^ of their relationships during the pandemic. Their responses connoted four different groups. First, those reporting no sexual activity tended to mention a lower quality of relationship, with relationships deteriorating due to lack of sexual activity and sometimes lack of trust that resulted from the challenge of distance associated with the COVID-19 context. Some males reported that they were irritable and angry at being denied sex; however, no physical abuse was reported. The men also tended to provide financially for their partners during this time.

*I had a girlfriend but she stays in Dodowa [a periurban town on the outskirts of LaNMMA] and because of the lockdown she couldn’t come out … The relationship was not stable, you will not have trust for your girlfriend. You will not even trust her if she says she is home, maybe she may be in another man’s room during the quarantine. The relationship was not stable. (Informally employed male, 20 years, no education*^5^*)*

Another young man, an apprentice, stated:

*Oh, I would get angry and I will tell her that I don’t like this thing [being denied sex] but she would also say something like she understands me and she would make it up to me and all those things … Ladies, they like hearing sweet words but we the boys the thing is in the touching, so if I don’t get that part, I would not be feeling the relationship … but I know if it wasn’t for the COVID-19, if I ask her right now, she would give me [sex]. (Male apprentice, 20 years, senior high school (SHS) education)*

A young female apprentice also gave her experience:

*He used to tell me that I am using COVID as an excuse not to have sex with him. And I will tell him that everyone is supposed to keep safe and I will not know who may have it, it could be me or it could be him. Because of his reactions, at a point, I wasn’t calling him and he also wasn’t calling me. (Female apprentice, 24 years, primary education)*

Second, four men and one woman reported their relationships were low quality despite some sexual contact with their partners during the lockdown. However, most of them reported infrequent encounters as well as issues of mistrust from being apart.

Third, the lack of sexual intimacy during the lockdown was also a time to communicate and bond with their partner. A few participants experienced high quality relationships at the time. More women stated this than the men. One male tertiary student reported enjoying appreciable time and bonding moments with his partner, which rather drew them closer during the lockdown period.

*Everything was good [with the relationship], it got to a point she didn't even want us to have sex again, she wanted us to continue the relationship like that [without sex] … It [the relationship] was good because we would always text. When I was coming [to the interview] I was even texting her. (Male tertiary student, 22 years, third year).*

A female apprentice also summed it up in this way:

*I think it [the bonding] is because we had more time to talk. On normal days we were both busy doing our own activities but during the lockdown we had time for each other and we could talk at any time without any distractions. (Female apprentice, 24 years, SHS education)*

Finally, the majority of women who were sexually active during that time and were living together with their partners faced no major issues in their relationships apart from “petty fights”. They generally reported high quality relationships at this time.

*During the lockdown, when we were not supposed to go out, fortunately I was with him … we had enough food to eat and we watched Netflix. We didn’t have any major argument except for the petty ones which we quickly resolved. (Informally employed female, 23 years, SHS education)*

Although restrictions were imposed on work, affecting some financially, young women who depended on partners stated they were still supported during the period. Young men also reported either explaining to their partners their inability to provide or were able to support them in some way. These emerging adults’ reports on their relationships connote varied effects from the stress of the lockdown and COVID-19 pandemic restrictions, thus, affirming some relationships while deteriorating others. The discussion below with a second year tertiary student sums up the negative experience of the lockdown on his relationship:

*I: … so in [during] the COVID time [lockdown period] during the period of March to May 2020 … how was your sexual relationship disrupted?*

*P: Oh, she lives very far from me so we couldn’t meet, we even planned [to meet] but it wasn’t successful … The communication went down, the vibe actually died … it’s reviving now.*

*I: So it [the lockdown] affected you in a way.*

*P: Yeah! Yeah! The sex life… It [the relationship’s quality] is not too strong now, it’s shaking. I was ignoring her because at a point I was not interested anymore. (Male tertiary student, 21 years, second year)*

### 3.3 Sexual activity

Out of the 12 participants who reported that they were sexually active, seven were males and five were females. Five of the participants were living together at the time of the lockdown. Five of these emerging adults reported having sex regularly during the period, and this was with people they considered as regular partners. One female respondent had sex with her ‘sugar daddy’^6^ while a male respondent had sex with an occasional partner he sometimes paid for sex. He mentioned the following:

*When I want sex, I have someone that I will call and ask how much the person will charge me. So when she comes I use my condom ….I did not have a girlfriend but I have someone that I will call and say I feel for sex today so how much will you charge me when you come; then we bargain. (Informally employed male, 20 years, no education)*

Other reports of sexual activity consisted of one or two sexual encounters during the March to May 2020 lockdown period.

*During the lockdown it was only once I had sex with her … During the lockdown I wanted her to come and stay with me but she was like her parents won’t agree and that made me mad and lashed out at her and she apologized to me and I let it go. (Male apprentice, 22 years, SHS education)*

*We didn’t expect it [the sex] to happen, it was like something spontaneous. Mostly we plan, I will tell her I will be coming so should I bring this or that [contraception] but with this [sexual encounter during the lockdown] we didn’t know it would happen. (Male apprentice, 20 years, SHS education)*

These findings imply some emerging adults made provision to spend the lockdown with their partners. Informal workers tended to report this. Others also found ways to meet despite the restrictions, and most of these young people reported infrequent sexual encounters. This was despite the harsh consequences during the initial three-week lockdown period where one male tertiary student stated: *“as I said [with the] lockdown, nobody is [was] supposed to go out … actually there is a police station down there. Police people were patrolling and when you go out, they will slap you, they will beat you” (Male tertiary student, 22 years, second year)*.

Male and female tertiary students as well as female apprentices reported the least amount of sexual encounters during this period – indicating that those in education as well as females in training were monitored and may not have had opportunities to engage in sexual activity unless they were able to leave home. Especially when their partners were also living at home. One tertiary student pointed out:

*Okay, so the stability of the relationship was okay but the quality of the relationship was distorted because at that time we were both in the house and our parents don’t let us come out and it’s someway [weird] because the more you meet the more you get to … the more you become closer, so the quality was getting distorted at some point … especially when the President added one week [to the lockdown period] (sucks his teeth loudly). (Male tertiary student, 22 years, second year)*

The lockdown restrictions and subsequent stresses affected emerging adults in the three socio-economic groupings differently. Some stayed apart and thus were sexually inactive, while others engaged in sex more frequently than before, due to their decision to cohabitate.

### 3.4 Contraceptive use/non-use and reproductive health issues

The sexually active participants reported using different modern and traditional contraception methods during the lockdown period. Male condoms (reported by 4 people) and emergency contraception (reported by 3 people) were the only modern methods mentioned. Traditional and folkloric methods were also used – two used the withdrawal method, one used the calendar method while a female participant used a method called “*kyem*” in the local language which involved squatting over a toilet and squeezing out the sperm immediately after sex.

Those using modern methods reported no barriers with accessing contraceptives. Some had condoms available in stock at home, while others had access to condoms from a relative's pharmacy or purchased it in bulk before the lockdown as indicated in the interviews below:

*I: Ok, how did you get the condoms? Did you buy them at pharmacy?*

*R: Yes, we buy from the pharmacy. We bought them when we were buying foodstuffs for lockdown. (Informally employed female, 23 years, SHS education)*

*R: Her uncle has a pharmacy, so she brought some when she came over during the lockdown. (Male apprentice, 20 years, SHS education)*

Regarding access to emergency contraceptive pills, one informal sector employee male mentioned regularly buying the ECP when at the pharmacy so he had some available during the lockdown.

*No, it [disruptions in access to contraception] never affected me. Anytime that I went to the pharmacy to get any medicine, I always buy the [EC] pill, maybe when I go to the pharmacy to buy say Dewormer, if I have enough money on me, I buy one Dewormer and one contraceptive. (Informally employed male, 23 years, SHS education).*

The findings from the interviews with the family planning providers indicate that access to provider administered modern contraceptive methods was restricted. Although family planning methods were available, the lockdown prevented people from getting access to services, thus decreasing patronage. The nurse at the private clinic noted:

*For the hospital, we had it [injectables] but no one was coming for it. Well, yeah, these injectables, they weren’t coming again at all. (Nurse, Private Clinic)*

*Yes, anyway, during the lockdown the patronage decreased but it didn't stop them at all [completely]. They were using it but it wasn't as frequent as now. (Nurse, Private Clinic).*

However, prior to the lockdown some individuals made provision by purchasing some commodities in advance, anticipating a period of engaging in some form of sexual activity. This corroborated some of the actions mentioned by the emerging adults:

*Before the lockdown, I think a day or two before the lockdown, most people were buying things, they were just buying things in excess. Condoms for instance, someone can buy like three or four packs, meanwhile a pack contains three [condoms]. (Medical Counter Assistant (MCA), Pharmacy)*

The provider at the pharmacy mentioned that some of their contraceptives were in short supply during the height of the pandemic. On the other hand, at the clinic, they had an existing stock of the products available throughout the lockdown period. This depicts young people's contraceptive behaviours and preferences in accessing modern contraceptive methods over-the-counter than from a health facility.

*During that time, we ran out of stock of most of them; I don’t know the reason why ….we weren’t having most of them. (MCA, Pharmacy)*

*The injectable, there was supply but the people were not coming. (Nurse, Private Clinic)*

Interestingly, amidst the price hikes in food, toiletries, personal protective equipment (PPE), detergents, sanitizer, and other essential commodities during the initial stages of the pandemic in Accra, prices of family planning services remained the same.

*I: What about the cost? Did you increase the cost of [family planning products] … ?*

*P: The price was the same (MCA, Pharmacy)*

*No, during the lockdown it wasn’t expensive but now it is somehow expensive. For the Lydia [emergency contraceptive pill] and the other ones too, some cost 7 cedis*^7^*but now 7.50 cedis or 8 cedis*^8^*(Nurse, Private Clinic)*

None of the emerging adults faced major reproductive health challenges. No reports of unintended pregnancies were mentioned despite some respondents’ regular engagement in sexual activity while using traditional methods such as withdrawal. This was the case for two young female informal workers. However, two emerging adults reported contracting sexually transmitted infections (STIs), candidiasis and gonorrhoea. One male respondent stated the following:

*R: She [his partner] had … Candidiasis and those things … and I got Gonorrhoea*

*I: You got Gonorrhoea?*

*R: Yeah*

*I: Ok, what did you do?*

*R: I bought medicine for myself (Informally employed male, 20 years, no education)*

The other informally employed 23-year-old female respondent reported that frequent use of the condom, their preferred method during the lockdown, caused her candidiasis.

Generally, restrictions during the lockdown period did not disrupt contraceptive use for those sexually active at the time. Emerging adults seemed to have adopted means of coping by relying on short-acting modern, traditional and folkloric methods they tended to use prior to the lockdown.

## 4 Discussion

Half of the respondents were sexually active and they reported different sexual and relationship encounters during the lockdown period. The lockdown sought to reduce the spread of the coronavirus in cities in the country, especially in Accra and Kumasi which reported the highest number of active cases and deaths (Kenu et al., 2020a; 2020b). As major urban centres were under lockdown and restrictions were placed on movement and social gatherings for some time, young people in sexually active relationships were affected in some way. Relationships and sexual activity were important considerations to these youth and some attempted to ensure these would thrive despite the psychological stress of the lockdown (Glanz et al., 2008). In line with the concept of coping, based on the psychological stress theory, some found ways to meet, move in together, engage in one-off sexual encounters, or stayed apart but continued communicating. Some appraised the quality of their relationships as diminished while others' stayed high. Young people's reports of verbal and emotional abuse due to lack of sex and infrequent sex was reported and conveys concern for some of these relationships that are being formed. These findings may reflect stress from the pandemic worsening already unhealthy relationships (Kenny, 2000; Krohne, 2002). Sexual and reproductive health education and counselling as well as self-efficacy may be the needed resources by emerging adults for coping, to ensure they are in safe relationships for the right reasons (Kenny, 2000).

Unlike relationship quality, the lockdown did not prove to be stressful on the contraceptive use of young adults who were sexually active during the period. Apart from emergency contraception, young people tended to use male-controlled and both-controlled methods that required negotiation between the couple (withdrawal, condoms, and calendar method). These findings indicate the different contraception choices among the sexually active urban youth during this critical period. They ensured they had access to their preferred contraceptive methods whether they would be effective in protecting against STIs and unplanned pregnancies or not. The acquiring of ECPs prior to the lockdown as opposed to other short-acting methods connotes a dependence of young people on its use outside of emergencies. Reasons for these have been indicated in other studies (L’Engle et al., 2011; Rokicki & Merten, 2018). Family planning providers also reported low patronage of provider administered modern methods during the lockdown period, although these commodities were available but perhaps not as accessible (Nation (Kenya Edition), 2021).

Solutions to improve access to modern contraceptive services may benefit some of the youth but those with a preference for traditional and folkloric methods must also be targeted with specialised interventions, including counselling. The use of short acting contraceptives and ECP prior to and during the lockdown also indicates the need for widespread government public health strategies to encourage the use of the spectrum of effective methods, including highly effective methods such as implants, injectables and intrauterine devices. Finally, governments need to consider contraceptive access in their response strategies to pandemics including that of COVID-19, as the findings of studies such as this indicate that challenges to access of the most effective modern contraceptive methods arose out of the lockdowns and restrictions on services not classified as essential (Bolarinwa et al., 2021; Cousins, 2020).

## 5 Conclusions

This study sought to explore the relationship quality, sexual activity and contraceptive behaviours and practices of emerging adults during the lockdown period caused by the COVID-19 pandemic in Accra, Ghana. The findings indicate for some emerging adults, relationship quality was largely dependent on sexual activity; thus, the lack of sex caused some to experience relationship instability, mistrust and non-communication during the lockdown period. Emerging adults engaged in frequent, occasional and no sex during the lockdown period with or without the use of effective contraception. Contraceptive use among those sexually active at the time included mostly short-acting modern methods and they relied on one of the many methods being used prior to the lockdown period. Traditional and folkloric method use was also preferred by some participants and was considered effective since no unintended pregnancies occurred. Improving the reproductive health behaviours of emerging adults during stressful and emergency situations, such as a pandemic, requires an understanding of their unique experiences.

## Funding

The study was funded by a grant (#INV-008737) from the 10.13039/100000865Bill & Melinda Gates Foundation, Seattle, WA to the 10.13039/501100011846International Union for the Scientific Study of Population (IUSSP), France to support the study of urban fertility and family planning.

## Author contributions

**Adriana Biney:** Conceptualization, Formal analysis, Funding acquisition, Investigation, Methodology, Supervision, Roles/Writing - original draft, Writing - review & editing; **Esinam Kayi:** Conceptualization, Investigation, Methodology, Writing - review & editing; **D. Yaw Atiglo:** Conceptualization, Formal analysis, Funding acquisition, Investigation, Methodology, Writing - review & editing; **Laud Sowah**: Conceptualization, Formal analysis, Data curation, Investigation, Methodology, Writing - review & editing; **Delali Badasu:** Project administration, Writing - review & editing; **Augustine Ankomah:** Project administration, Writing - review & editing.

## Data statement

The transcripts used for this study are available upon request by contacting the corresponding author - abiney@ug.edu.gh.

## Declaration of competing interest

The authors declare that they have no known competing financial interests or personal relationships that could have appeared to influence the work reported in this paper.

## Footnotes

1 In this study we define the lockdown period as during the three week “official lockdown” in Accra from the end of March to mid-April 2020 and we also include the critical period from mid-April to late May 2020 when the COVID-19 pandemic was still at its peak. Social and religious gatherings comprising more than 25 people were prohibited; and there was still much fear and stigma associated with the novel coronavirus.

2 None of the 23 emerging adults in relationships at the time of the lockdown period were 18 years; hence the age range of 19–24 years.

3 Using an exchange rate in January 2021 of approximately GHS5.85 to US$1.

4 To determine relationship quality, we asked participants to describe whether their relationship was stable, whether there was abuse, arguments, fights, regular communication, and trust between them.

5 We report the socio-economic group, sex, age and highest educational level attained for each respondent. For tertiary students we report their undergraduate year.

6 A sugar daddy is a male sexual partner of a female. She is usually far younger than the man and she enjoys financial and other benefits from the relationship, most of the time in exchange for sex.

7 The exchange rate ranged between GHS5.47 and GHS5.75 to US$1 between March and May, so the cost during the lockdown and critical COVID times would have been between US$1.21 and US$1.28.

8 The exchange rate at the time of the interview in February 2021 was about GHS5.75 to US$1 – so the cost was approximately US$1.39.
